# Lumbar spine MRI versus non-lumbar imaging modalities in the diagnosis of sacral insufficiency fracture: a retrospective observational study

**DOI:** 10.1186/s12891-018-2189-1

**Published:** 2018-07-25

**Authors:** Yoon Yi Kim, Bo Mi Chung, Wan Tae Kim

**Affiliations:** Department of Radiology, Veterans Health Service Medical Center, 53, Jinhwangdo-ro 61-gil, Gangdong-gu, Seoul, 05368 Republic of Korea

**Keywords:** Sacral insufficiency fracture, Sacrum, MRI, Lumbar spine MRI

## Abstract

**Background:**

Sacral insufficiency fractures (SIFs) are a common cause of lower back pain in the elderly. However, because clinical symptoms are frequently vague and nonspecific and can mimic lumbar spine pathologies, initial imaging in SIF patients is frequently targeted at the lumbar spine rather than the sacrum, resulting in delayed diagnosis. The purpose of this study is to show the proportions of modalities used in diagnosing SIF in practice and to compare the clinical and imaging features of SIF diagnosed by lumbar spine MRI (L-spine MRI) with those diagnosed by non-lumbar imaging modalities (bone scan, pelvic bone CT, pelvis MRI).

**Methods:**

Forty-two patients with SIF were enrolled in this study. SIFs diagnosed by L-spine were assigned to group 1 and SIFs diagnosed by non-lumbar imaging modalities (bone scan, pelvic bone CT, pelvis MRI) were assigned to group 2. The clinical and imaging features of SIFs were assessed and compared between two groups.

**Results:**

SIF were more commonly diagnosed by L-spine MRI (group 1: *n* = 27, 64.3%) than non-lumbar imaging modalities (group 2: *n* = 15, 35.7%), which was comprised of pelvic bone CT (*n* = 6, 14.3%), bone scan (*n* = 5, 11.9%), and pelvis MRI (*n* = 4, 9.5%). Lower back pain, radiating pain and comorbid other causes of pain were more frequently identified in group 1. Fracture involving bilateral sacral ala with horizontal component was the most common shape and S2 being the most commonly involved horizontal component, without significant difference between two groups.

**Conclusion:**

SIFs are more commonly diagnosed by L-spine MRI than non-lumbar imaging modalities, because of symptoms that mimic lumbar spine pathology and variable comorbid causes of pain. To know that L-spine MRI commonly reveal SIF and to be familiar with SIF features on L-spine MRI would help increase sensitivity in detecting this commonly underrecognized entity and achieve earlier and more appropriate management.

## Background

Sacral insufficiency fractures (SIFs) are a common cause of lower back pain in the elderly, with a mean age between 70 and 75 years in most studies [1–3]. They were first described as a clinical entity in 1982 by Lourie, and the awareness and reports of this entity have increased with the increasing number of elderly patients [4, 5]. American College of Radiology (ACR) appropriateness criteria recommend radiography as the first imaging study when SIF is suspected. When radiography is negative, the next imaging study recommended is pelvis MRI without intravenous contrast or bone scan, with reported high sensitivities [6]. However, because clinical symptoms are frequently vague and nonspecific and can mimic lumbar spine pathologies, initial imaging in SIF patients is frequently targeted at the lumbar spine rather than the sacrum, resulting in delayed diagnosis [1, 6–8].

Although many authors suggest radiologists and clinicians to be aware of this entity in interpreting L-spine MRI in old age patients [1, 9], there has been no study that revealed the proportion of L-spine MRI among the modalities used in diagnosing SIFs or analyzed the difference in clinical and imaging characteristics of SIFs between the L-spine MRI and the non-lumbar imaging modalities.

The purpose of this study is to show the proportions of modalities used in diagnosing SIF in practice and to compare the clinical and imaging features of SIF diagnosed by L-spine MRI with those diagnosed by non-lumbar imaging modalities (bone scan, pelvic bone CT, pelvis MRI).

## Methods

### Study population

**Fig. 1:**
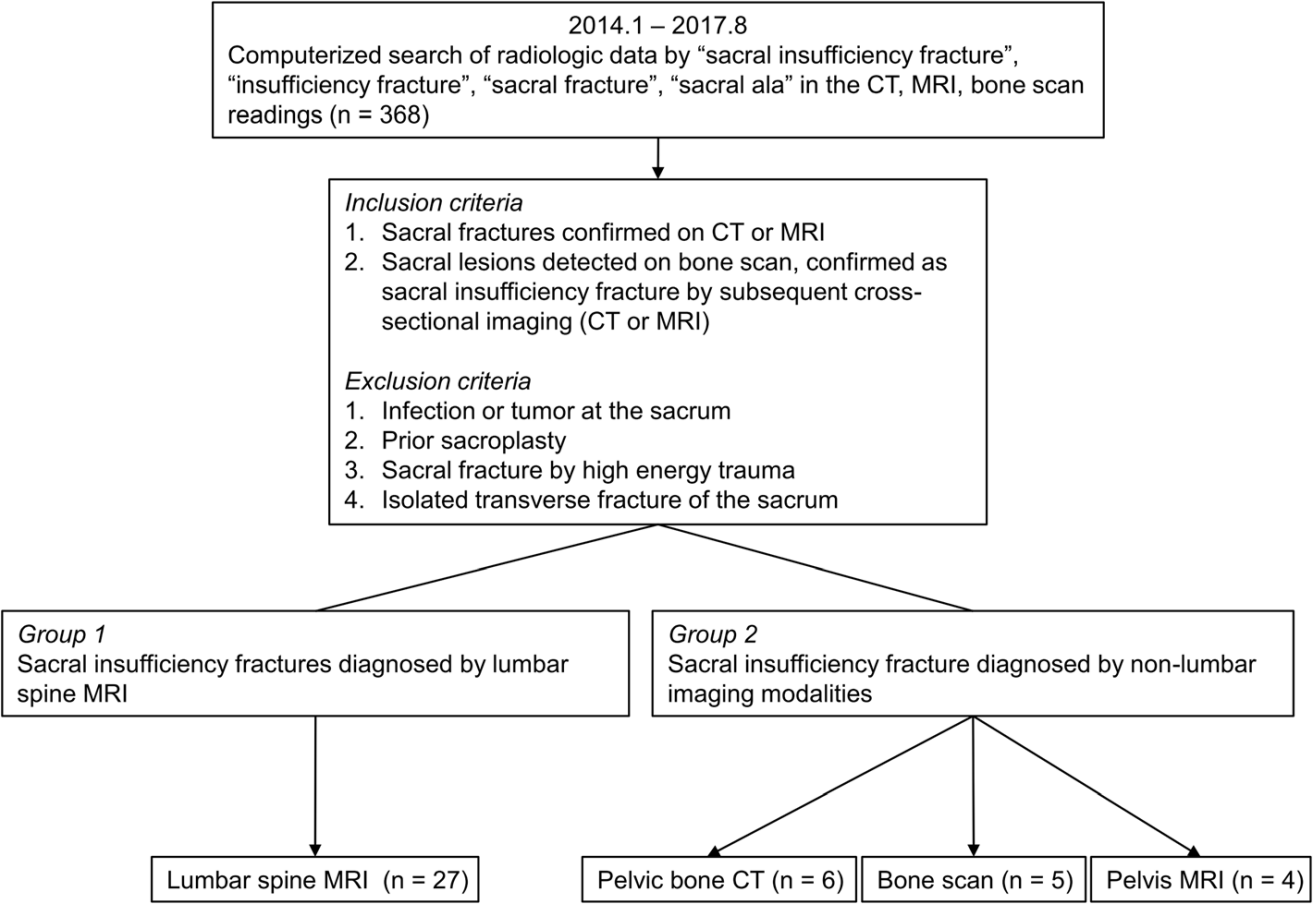
Flow chart of the inclusion process of study group

This study was approved by the institutional review board, and informed consent was waived due to the retrospective nature of the study. Figure 1 is a flowchart that summarizes the inclusion process in this study. A picture archiving and communication system (PACS) search was conducted using the keywords “sacral insufficiency fracture,” “insufficiency fracture,” “sacral fracture,” “sacral ala” in the CT, MRI, and bone scan readings at our hospital between January 2014 and August 2017 (*n* = 368). To be enrolled in this study, patients were required to have sacral fracture confirmed on CT or MRI (*n* = 65). We excluded patients with infection or tumor at the sacrum (*n* = 5), prior sacroplasty (*n* = 1), sacral fracture by high energy trauma (*n* = 4), or isolated transverse fracture of the sacrum (*n* = 13). Finally, 42 patients (15 men, 27 women; mean age, 78.83 years; age range, 59–94 years) with SIF were enrolled in this study. Among the 42 patients, 7 patients had two imaging modalities that demonstrated SIF. In 5 patients, SIFs were initially detected by bone scan and confirmed on cross-sectional imaging (4 patients with CT, 1 patient with MRI). One patient underwent L-spine MRI and subsequent pelvis MRI, the other patient initially underwent pelvic bone CT and then bone scan. The interval between two imaging modalities ranged from 1 to 37 days. SIFs initially detected and diagnosed by L-spine were assigned to group 1 (*n* = 27). SIFs initially detected by non-lumbar imaging modalities (bone scan, pelvic bone CT, pelvis MRI) were assigned to group 2 (*n* = 15).

All patients were confirmed as having SIF based on CT or MRI findings. A retrospective review of these patients was conducted by two musculoskeletal radiologists with 2 and 13 years of experience in musculoskeletal image interpretation. An SIF was diagnosed on CT when sagittally oriented fracture lines with or without bony callous were evident at the sacral ala [1, 9]. On MRI, SIF was confirmed by detecting bone marrow edema at the sacral ala, demonstrated as a hypointense area on T1-weighted image (T1-WI) or a hyperintense area with a hypointense fracture line on fat-suppressed T2-weighted image (T2-WI) [1, 9]. In cases initially detected by bone scan, subsequent cross-sectional imaging (either CT or MRI) to confirm the diagnosis was required for patient inclusion in this study [1, 9].

The electronic medical record was reviewed for patient age, gender, body mass index (BMI), presence or absence of osteoporosis by bone mineral density (BMD), low-energy trauma history, operation history at spine or hip, past medical history, and chief complaint. The BMD and BMI were available for 23 and 33 out of a total of 42 patients, respectively. Through the review of medical records and all the imaging studies performed around the time of detection of SIF, we recorded the presence or absence of vertebral compression fractures and other causes of pain including acute vertebral compression fracture, spinal stenosis, insufficiency fracture of pelvic bone other than the sacrum, and acute hip fracture.

### Image acquisition

MR images were obtained using either 1.5-T (Signa, GE) or 3-T (Magnetom Skyra, Siemens) scanners. The protocols varied somewhat depending on the machines or the clinician’s request at our institution, but most patients had a combination of the following sequences: sagittal T2-WI (section thickness 4 mm; section gap 0.4–0.5 mm; matrix 448–512 × 256–448; TR 2920–3630; TE 88–114), sagittal T1-WI (section thickness 4 mm; section gap 0.4–0.5 mm; matrix 448–512 × 256–369; TR 460–652; TE 7–11), axial T2-WI (section thickness 4 mm; section gap 0.4–0.5 mm; matrix 320–448 × 202–224; TR 3590–4784; TE 95–104), and axial T1-WI (section thickness 4 mm; section gap 0.4–0.5 mm; matrix 320–448 × 202–224; TR 603–821; TE 9–14). Seventeen patients had additional sequences using fat suppression techniques including sagittal fat-suppressed T2-WI (slice thickness 4 mm; section gap 0.5 mm; matrix 352 × 224; TR 2897; TE 90) on 1.5-T and Dixon sequence water image (slice thickness 4 mm; section gap 0.4 mm; matrix 382 × 307; TR 2880–3720; TE 93) on 3-T MRI. Twenty-two patients had coronal T2-WI (section thickness 4 mm; section gap 0.4–0.5 mm; matrix 448–512 × 224–314; TR 2500–3330; TE 88–114).

The area of coverage was T10 to S3 on sagittal images, and bilateral neural foramina and bilateral sacroiliac joints were included on parasagittal images. On axial imaging, disc spaces including endplates were included for at least four levels of L2/3, L3/4, L4/5, and L5/S1. In eight patients, axial images of the sacrum to S3 level were also acquired. On coronal images, vertebral bodies and the spinal canals of lumbar spines were included, which covered variable portion of sacrum according to the degree of sacral lordosis.

CT examinations were performed using 64-channel (Sensation 64, Siemens) CT systems. The scanning parameters were tube voltage of 120 KV, quality reference tube current of 150 mA, a rotation time of 1 s, a pitch of 0.9, a collimation of 0.6 mm, and a section thickness/reconstruction interval of 2 mm. In addition to axial images, coronal and sagittal images were reformatted with section thickness/reconstruction interval of 2 mm. The field of view were variable depending on the clinician’s request, but invariably included the sacrum from S1 down to below the lesser trochanters.

### Image analysis

**Fig. 2:**
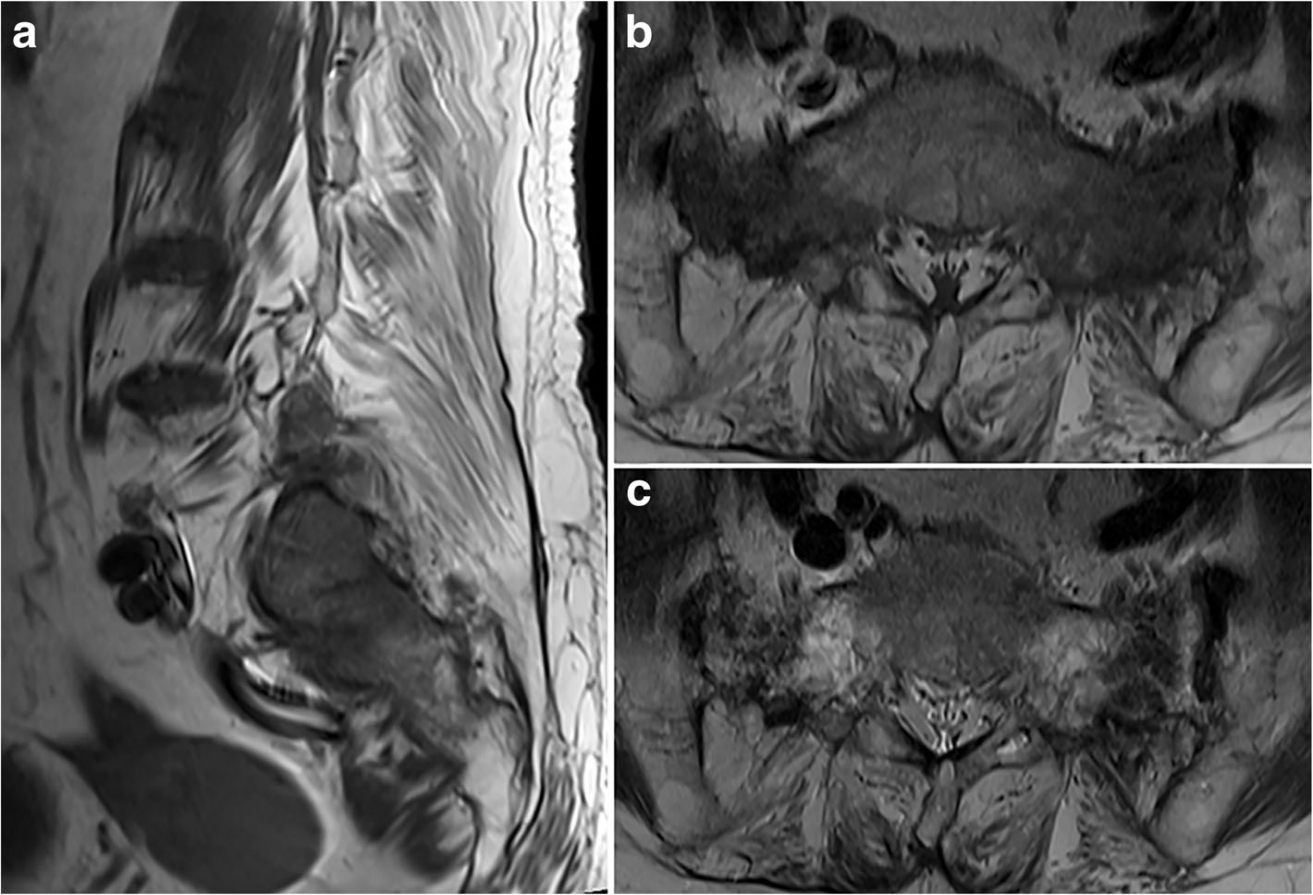
A 72-year-old woman with sacral insufficiency fracture (SIF) involving the bilateral sacral alae with horizontal component. **a** Sagittal T1-weighted MR image shows diffuse signal alteration that suggests moderate bone marrow edema at the left sacral ala. **b** Axial T1-weighted MR image at the proximal S1 level shows moderate bone marrow edema at both sacral alae. **c** Axial T2-weighted MR image of the same level shows hypointense fracture lines at both sacral alae

**Fig. 3:**
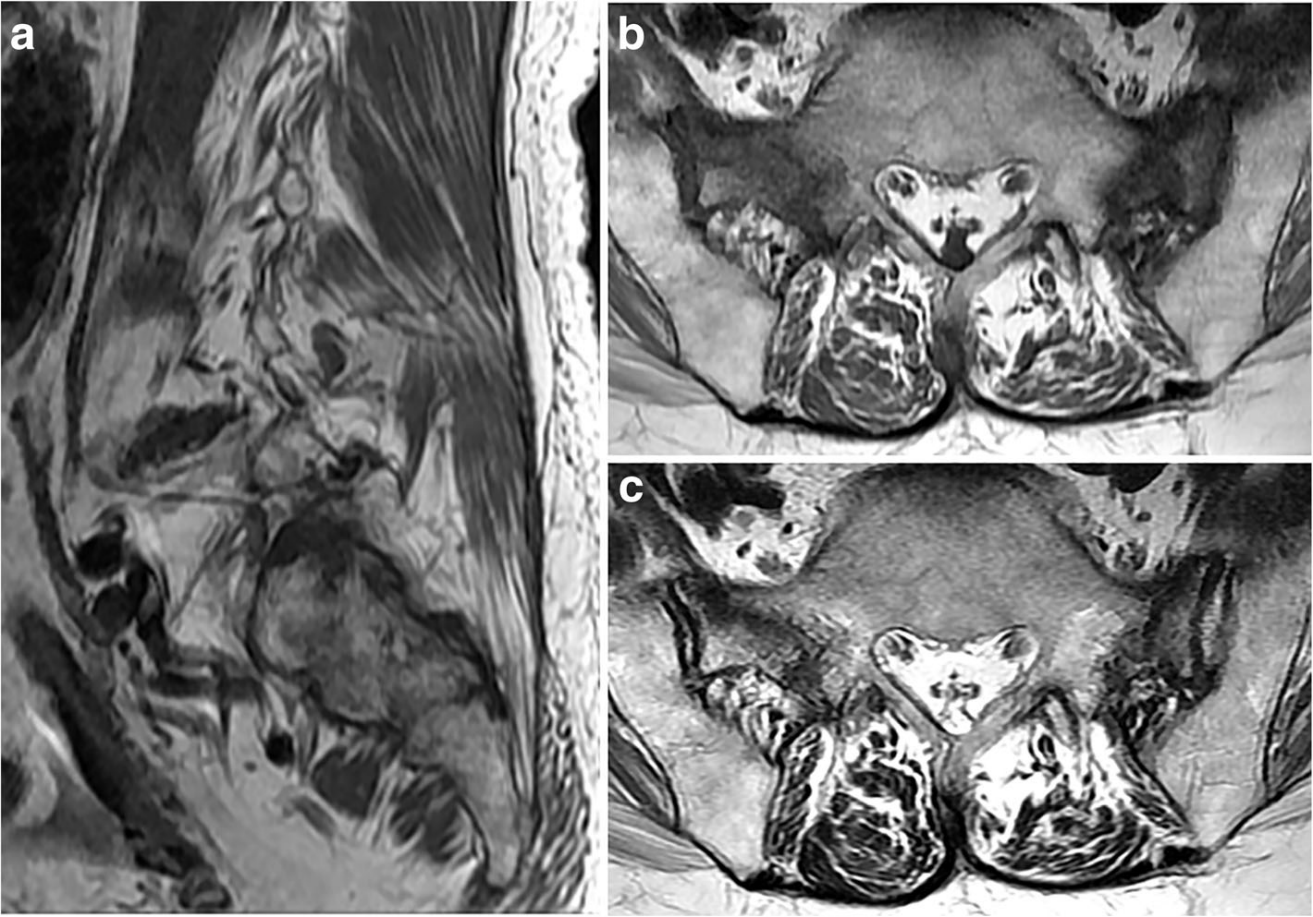
A 68-year-old woman with SIF involving the bilateral sacral alae. **a** Sagittal T1-weighted MR image shows irregular signal alteration suggesting mild bone marrow edema at the left sacral alae. **b** Axial T1-weighted MR image at the proximal S1 level shows hypointense fracture lines with surrounding bone marrow edema at both sacral alae. **c** Axial T2-weighted MR image at the same level shows hypointense fracture line

**Fig. 4:**
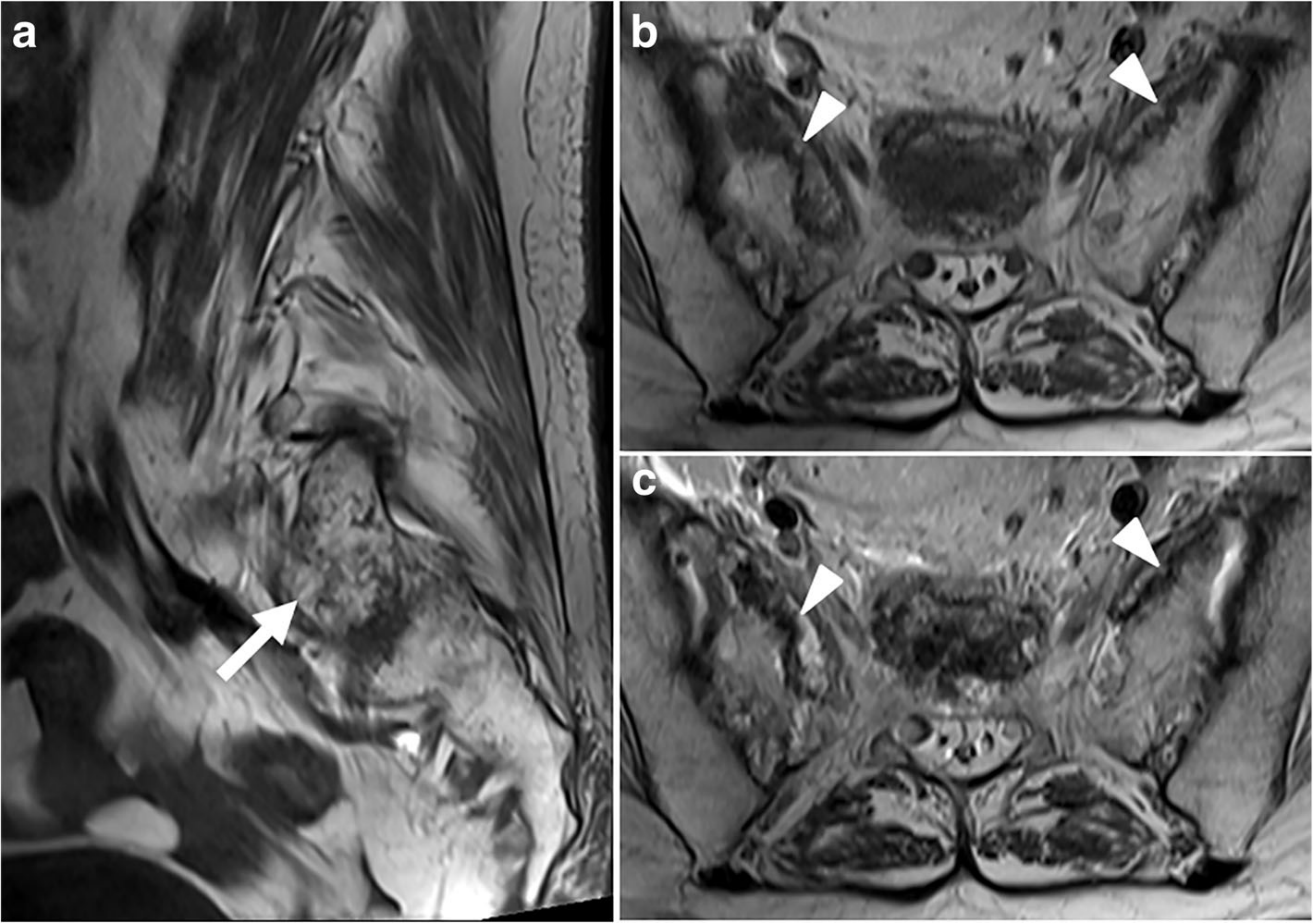
An 81-year-old woman with SIF involving the bilateral sacral alae with a horizontal component (not shown). **a** Sagittal T1-weighted MR image shows hypointense streaks at the right sacral ala (arrow). **b** and **c**, Axial T1-weighted (**b**) and T2-weighted (**c**) MR images at the distal S1 level show hypointense fracture lines at both sacral alae with minimal surrounding bone marrow edema (arrowheads)

The MR images were evaluated using a PACS workstation. The evaluation of imaging features was performed by consensus of two radiologists with 2 and 13 years of experience in musculoskeletal MRI interpretation (BM C [reader 1] and WT K [reader 2], respectively). We assessed and classified the shape of all SIFs into four groups; vertical fracture at unilateral sacral ala (U), vertical fractures at bilateral sacral alae (B), vertical fracture at unilateral sacral ala with horizontal component (UH), and vertical fractures at bilateral sacral alae with horizontal component (BH). The level of horizontal component fracture was recorded. The degree of bone marrow edema around vertical fracture of sacral ala shown on T1-WI in the sagittal plane were recorded in group 1 and categorized as ‘moderate’ when there was diffuse bone marrow edema with barely visible normal fatty marrow (Fig. 2). The degree was recorded as ‘mild’ when bone marrow edema occupied between one-third and two-thirds of the diseased area (Fig. 3) and ‘minimal’ when only hypointense streaks or nodular lesions were seen (Fig. 4). Six months after the initial imaging analysis, reader 1 again evaluated the degree of bone marrow edema, avoiding recall bias. The reader 3 (YY K) independently rated the bone marrow edema with the same criteria. The interobserver and intraobserver agreement was evaluated.

### Statistical analysis

To assess the differences in the clinical and imaging features between group 1 and 2, the Chi-square test, Fisher’s exact test, Student’s t test, and Mann–Whitney test were utilized for analysis. Agreement regarding the degree of bone marrow edema was statistically compared with the kappa (κ) statistics. κ values of interobserver and intraobserver agreement was assigned as follows: less than 0.20, poor; 0.21–0.40, fair; 0.41–0.60, moderate; 0.61–0.80, good; and more than 0.81, excellent [10]. A *p* value less than 0.05 was considered statistically significant. All analyses were performed with IBM SPSS 20 (IBM Software Inc.).

## Results

Out of 42 patients, 27 SIFs were diagnosed by L-spine MRI and assigned to group 1 (64.3%). Fifteen SIFs were detected and diagnosed by non-lumbar imaging modalities and were assigned to group 2 (35.7%). Group 2 was comprised of pelvic bone CT (*n* = 6, 14.3%), bone scan (*n* = 5, 11.9%), and pelvis MRI (*n* = 4, 9.5%).

The clinical features of patients are summarized in Table 1. In terms of chief complaint, lower back pain and radiating pain were more frequently identified in group 1, whereas hip pain was more commonly associated with group 2. Other causes of pain were present in most patients (*n* = 31, 73.8%) and were significantly more common in group 1. The most common other cause of pain was spinal stenosis followed by acute vertebral compression fracture in group 1 and acute hip fracture in group 2. History of malignancy was more common in group 2. No significant difference between the two groups was noted in other clinical features.

**Table 1:** Clinical Features of Sacral Insufficiency Fractures

	Group 1 (*n* = 27)	Group 2(*n* = 15)	Total (*n* = 42)	*P* value
Age (years)	79.5±7.8	77.7±12.1	78.8±9.3	0.599
Male:Female	12:15	3:12	15:27	0.180
Osteoporosis	8 (n = 13)	9 (*n* = 10)	17 (*n* = 23)	0.179
BMI	22.5±3.5	23.0 ±3.5	22.7±3.4	0.690
Trauma history	13	8	21	1.000
Prior surgery (hip, spine)	7	4	11	1.000
Vertebral compression fracture	18	8	26	0.511
Past medical history				
Diabetes	4	1	5	0.639
Hypertension	10	4	14	0.734
Chronic renal disease	2	1	3	1.000
Malignancy	2	6	8	0.016
Chief complaint				
Lower back pain	24	2	26	0.000
Radiating pain	10	0	10	0.007
Hip pain	2	10	12	0.000
Other cause of pain	23	8	31	0.034
Acute vertebral compression fracture	9	2	11	0.273
Spinal stenosis	17	0	17	0.000
Other PIF	3	2	5	1.000
Acute hip fracture	1	4	5	0.047

*BMI* body mass index, *PIF* pelvic insufficiency fracture

Values in parenthesis for osteoporosis represent the number of patients with available bone mineral density (BMD) results

The imaging features of SIFs are summarized in Table 2. The BH shape was the most common (*n* = 28, 66.7%), followed by UH (*n* = 6, 14.3%), B (*n* = 5, 11.9%), and U (*n* = 3, 7.1%) shapes. The most commonly involved horizontal component was S2 (*n* = 14, 41.2%). No statistically significant difference was noted between the two groups regarding fracture shape or the level of the horizontal component. On sagittal T1-WI, bone marrow edema around vertical fracture most commonly showed moderate (*n* = 24, 49%) degree, followed by mild (*n* = 17, 34.7%) or minimal (n = 6, 16.3%) degrees. The interobserver agreement between the first reading and reader 3 was good (κ = 0.693). The intraobserver agreement between the two readings by reader 1 was excellent (κ = 0.831).

**Table 2:** Imaging Features of Sacral Insufficiency Fractures

	Group 1 (*n* = 27)	Group 2(*n* = 15)	Total (*n* = 42)	*P* value
SIF shape				0.102
U	2	1	3 (7.1%)	
B	1	4	5 (11.9%)	
UH	3	3	6 (14.3%)	
BH	21	7	28 (66.7%)	
H component level				0.158
S1	1	2	3 (8.8%)	
S1,2	2	0	2 (5.9%)	
S2	11	3	14 (41.2%)	
S2,3	3	0	3 (8.8%)	
S3	7	5	12 (35.3%)	

*SIF* sacral insufficiency fracture, *U* unilateral sacral ala, *B* bilateral sacral alae, *UH* unilateral sacral ala with horizontal component, *BH* bilateral sacral alae with horizontal component, *H* horizontal

## Discussion

Insufficiency fractures occur when normal stresses are applied to bone with decreased density. They occur most commonly in the pelvis, including the sacrum, followed by the proximal femur and the vertebral bodies. SIFs commonly affect elderly women with osteoporosis [1, 5, 11, 12]. Antecedent trauma is not identified in two-thirds of patients and, when present, is usually minor [1, 2, 13]. Clinical features including age, gender, presence of osteoporosis, and trauma history were comparable with previous reports and showed no significant difference between the two groups.

Various modalities are in use to diagnose SIF in daily practice, and this is the first study to show the proportion of modalities and analyzed the clinical and imaging features. Although sacrum is not routinely covered completely on L-spine MRI, our study revealed that L-spine MRI was the most common modality that detected SIF (*n* = 27, 64.3%), followed by pelvic bone CT (*n* = 6, 14.3%), bone scan (*n* = 5, 11.9%), and pelvis MRI (*n* = 4, 9.5%). In 73.8% of all patients, other comorbid causes of pain were present. The advanced age of SIF patients and the risk factors represented by osteoporosis would have contributed to this result. Other cause of pain was significantly more common in group 1 and were mainly comprised of spinal stenosis and acute vertebral compression fracture. This result partly explains the frequent chief complaints of lower back pain and radiating pain in group 1. The choice of L-spine MRI seems to be effective to find both lumbar spine pathology and SIF. Acute hip fracture and hip pain complaints was more prevalent in group 2, and it explains why these SIFs were diagnosed by non-lumbar imaging studies. History of malignancy was more common in group 2, which was associated with the SIFs detected in the screening bone scan for bone metastasis in patients with malignancy history.

SIFs most commonly involve the bilateral or unilateral sacral alae, lateral to the neural foramina and medial to the sacroiliac joints. There can also be a horizontal component to the fracture through the sacral bodies [1, 14]. BH was the most common shape of SIF, and S2 was the most common horizontal level in our study, which was in accordance with the literature [14–16]. Authors expected that the fracture shape might be related with clinical presentation and the choice of modality but there was no statistically significant difference between the two groups. We excluded isolated horizontal fracture of the sacrum in this study because it is controversial whether this is really an insufficiency fracture versus a fracture caused by minor trauma [14, 17]. Linstrom et al. introduced isolated horizontal fracture as an atypical SIF shape in cases with unusual sacral stress patterns, such as extreme amounts of sacral lordosis. However, more commonly the horizontal component seems to develop at a later stage after loss of sacral alar support, which causes the entire weight of the upper body to be longitudinally transferred down the central portion of the sacral bodies [14, 18].

Bone scan, CT, and MRI were utilized to diagnose SIF in our study. Bone scan is one of the most sensitive examinations for the detection of SIF and regarded as gold standard for detecting insufficiency fractures for many years. Some authors have suggested that the H-shaped (Honda or butterfly) sacral pattern could be considered diagnostic in the correct clinical setting, especially if there are no other sites of abnormal uptake and no history of primary malignancy. But this characteristic pattern is seen in only 20 to 40% of patients and variations in the pattern of radiopharmaceutical activity could be seen [6, 11]. There also have been case reports of isolated metastases presenting as unilateral sacral uptake [16]. Therefore, we included only cases that were confirmed by cross-sectional imaging when bone scan suggested the possibility of SIF. CT is less sensitive for the detection of SIFs than bone scan or MRI, with a reported sensitivity between 60 and 75%, and is not typically used for first or second-line imaging tool for work up of insufficiency fractures. However, CT may offer adjunct role to confirm inconclusive or equivocal findings on bone scan or MRI [1, 6]. MRI can detect SIF very sensitively like bone scan, with higher specificity than bone scan. MRI can usually differentiate insufficiency fracture from pathologic fracture due to tumor infiltration [1]. Recent literature favors MRI for making early diagnosis of insufficiency fractures at pelvic region [1, 6, 19]. The fat suppressed images are especially sensitive for the detection of early bone marrow edema and coronal imaging of sacrum are recommended to be included in suspected cases [1, 20]. Gupta et al. reported that addition of coronal short tau inversion recovery sequence to the L-spine MRI enabled them to detect significant findings in 6.8% of patients, including SIF or sacroiliitis [21].

Although fat suppressed images can more sensitively detect bone marrow edema and coronal imaging of sacrum can better demonstrate vertically oriented fracture line, it is difficult to suspect SIF on physical examination and add these sequences before imaging, due to the ambiguity of SIF as discussed ahead [1, 20]. Therefore, we evaluated imaging features of SIF based on sagittal T1-WI, which is generally available sequence in most L-spine MRI studies. Getting used to the MRI findings with a scan range and sequences of routine L-spine MRI would help to reduce the underrecognized SIF. The vertical sacral ala fracture most commonly showed moderate bone marrow edema (49%), followed by mild and minimal degrees. The vertical fracture of sacral ala with moderate bone marrow edema would be readily detectable. In addition, based on our result, careful detection of irregular or reticular pattern could enable the early diagnosis of the otherwise overlooked SIF.

There are several limitations in our study. First, MRI protocols were variable because of the retrospective nature of this study. Second, the distal sacrum was not entirely covered because the routine L-spine MRI covered distally to S3 body in our institution. However, the horizontal component of SIF involved S1, 2 or 3 not only in group 1 but also in group 2 which fully covered sacrum. Third, this is a single-center study. The choice of imaging modality and protocol of L-spine MRI could vary according to the clinicians and institutions. Multicenter, prospective studies are needed for further verification. Lastly, the number of SIFs diagnosed by bone scan could be underestimated because only cases confirmed on cross-sectional images were included in this study.

## Conclusions

In conclusion, SIFs are more commonly diagnosed by L-spine MRI than non-lumbar imaging modalities in practice, because SIF frequently mimics lumbar spine pathology due to ambiguous symptoms and variable comorbid causes of pain. Knowing that L-spine MRI commonly reveal SIF and to be familiar with SIF features on L-spine MRI would help radiologists and clinicians to sensitively diagnose this commonly underrecognized entity and achieve earlier and more appropriate management.

## Abbreviations

BMD: Bone mineral density
BMI: Body mass index
CT: Computed tomography
L-spine MRI: Lumbar spine MRI
MRI: Magnetic resonance imaging
SIF: Sacral insufficiency fracture
